# Factor Models as an Important Tool in Creating the Effective Quality Management System in Psychiatric Institutions

**DOI:** 10.1192/j.eurpsy.2025.1617

**Published:** 2025-08-26

**Authors:** V. Mitikhin, T. Solokhina, D. Oshevsky

**Affiliations:** 1 Mental Health Research Centre, Moscow, Russian Federation

## Abstract

**Introduction:**

Medical staff is one of the main participants in psychiatric care quality management system. At the same time, it is not enough to ensure high level of staff qualifications only. It is also necessary to determine staff satisfaction with their professional activities, levels of staff well-being and of professional burden. That’s why, using factor analysis in the process of identification of factors affecting these parameters of psychiatric staff is an important task.

**Objectives:**

To work out factor models determining the main components affecting psychiatric staff professional burden and satisfaction of staff with provided psychiatric care; to justify proposals for improving medical staff professional activities and quality of care.

**Methods:**

Adapted questionnaire «Assessment of the burden of psychiatric staff working in psychiatric institution» (WHO, 1994); Questionnaire «Assessing the satisfaction with quality of care by medical staff of psychiatric institution» (Solokhina et al., 2014). Factor analysis (principal component analysis with quatrimax rotation and factor selection according to the Cattell criterion) was used in the IBM SPSS Statistics 27 software environment. The study involved 73 nurses (age 44,55±11,56) of Moscow psychiatric hospitals № 1 and № 4. The nurses were included in the analyses as the most representative staff category closely contacting with patients.

**Results:**

Using factor analysis, a model identifying the most significant components affecting of the professional burden of the nurses and reflecting 50.9% of the sample variance was worked out. The parameters include: “physical and emotional problems”, “interpersonal problems”, “intrapersonal problems”, “stigmatization”, “experiencing difficult situations related to patients” (16.86%, 11.67%, 9.52%, 6.61%, 6.34 of the sample variances correspondingly).

The factor model of nurse’s satisfaction with psychiatric institutions activities includes “organizational” (33.66%), “procedural” (7.44%), “logistical” (6.92%), “support” (5.14%), “alternative forms of assistance” (5.05%) factors, which in total make up 58.8% of the sample variance.

**Conclusions:**

Factor models are a powerful tool which permits to analyze complex information and to identify factors affecting important indicators in a quality management system. Therefore, factor analysis should be carried out regularly in order to prevent different risks of care quality violation. It is also important to introduce psychological support to medical staff and to improve team methods of work.

**Disclosure of Interest:**

None Declared

